# Class effects of SGLT2 inhibitors in mouse cardiomyocytes and hearts: inhibition of Na^+^/H^+^ exchanger, lowering of cytosolic Na^+^ and vasodilation

**DOI:** 10.1007/s00125-017-4509-7

**Published:** 2017-12-02

**Authors:** Laween Uthman, Antonius Baartscheer, Boris Bleijlevens, Cees A. Schumacher, Jan W. T. Fiolet, Anneke Koeman, Milena Jancev, Markus W. Hollmann, Nina C. Weber, Ruben Coronel, Coert J. Zuurbier

**Affiliations:** 1 Laboratory of Experimental Intensive Care and Anaesthesiology, Department of Anaesthesiology, Academic Medical Center, University of Amsterdam, Meibergdreef 9, 1105 AZ Amsterdam, the Netherlands; 2 Department of Clinical and Experimental Cardiology, Academic Medical Center, University of Amsterdam, Amsterdam, the Netherlands; 3 Department of Medical Biochemistry, Academic Medical Center, University of Amsterdam, Amsterdam, the Netherlands

**Keywords:** Cardiac, Diabetes, Heart failure, Na^+^/H^+^ exchanger, SGLT2i, Sodium, Vasodilation

## Abstract

**Aims/hypothesis:**

Sodium–glucose cotransporter 2 (SGLT2) inhibitors (SGLT2i) constitute a novel class of glucose-lowering (type 2) kidney-targeted agents. We recently reported that the SGLT2i empagliflozin (EMPA) reduced cardiac cytosolic Na^+^ ([Na^+^]_c_) and cytosolic Ca^2+^ ([Ca^2+^]_c_) concentrations through inhibition of Na^+^/H^+^ exchanger (NHE). Here, we examine (1) whether the SGLT2i dapagliflozin (DAPA) and canagliflozin (CANA) also inhibit NHE and reduce [Na^+^]_c_; (2) a structural model for the interaction of SGLT2i to NHE; (3) to what extent SGLT2i affect the haemodynamic and metabolic performance of isolated hearts of healthy mice.

**Methods:**

Cardiac NHE activity and [Na^+^]_c_ in mouse cardiomyocytes were measured in the presence of clinically relevant concentrations of EMPA (1 μmol/l), DAPA (1 μmol/l), CANA (3 μmol/l) or vehicle. NHE docking simulation studies were applied to explore potential binding sites for SGTL2i. Constant-flow Langendorff-perfused mouse hearts were subjected to SGLT2i for 30 min, and cardiovascular function, O_2_ consumption and energetics (phosphocreatine (PCr)/ATP) were determined.

**Results:**

EMPA, DAPA and CANA inhibited NHE activity (measured through low pH recovery after NH_4_^+^ pulse: EMPA 6.69 ± 0.09, DAPA 6.77 ± 0.12 and CANA 6.80 ± 0.18 vs vehicle 7.09 ± 0.09; *p* < 0.001 for all three comparisons) and reduced [Na^+^]_c_ (in mmol/l: EMPA 10.0 ± 0.5, DAPA 10.7 ± 0.7 and CANA 11.0 ± 0.9 vs vehicle 12.7 ± 0.7; *p* < 0.001). Docking studies provided high binding affinity of all three SGLT2i with the extracellular Na^+^-binding site of NHE. EMPA and CANA, but not DAPA, induced coronary vasodilation of the intact heart. PCr/ATP remained unaffected.

**Conclusions/interpretation:**

EMPA, DAPA and CANA directly inhibit cardiac NHE flux and reduce [Na^+^]_c_, possibly by binding with the Na^+^-binding site of NHE-1. Furthermore, EMPA and CANA affect the healthy heart by inducing vasodilation. The [Na^+^]_c_-lowering class effect of SGLT2i is a potential approach to combat elevated [Na^+^]_c_ that is known to occur in heart failure and diabetes.

**Electronic supplementary material:**

The online version of this article (10.1007/s00125-017-4509-7) contains peer-reviewed but unedited supplementary material, which is available to authorised users.

## Introduction

Sodium–glucose cotransporter 2 (SGLT2) inhibitors (SGLT2i) are a new class of type 2 diabetic agents that control plasma glucose levels by inhibiting reabsorption of glucose and sodium in the proximal tubules of the kidney [1]. Of the several different SGLT2i, empagliflozin (EMPA) and canagliflozin (CANA) have shown cardiovascular benefits in type 2 diabetic individuals, with a remarkable 35% and 32% reduction, respectively, in hospitalisation for heart failure [2, 3]. These effects could not be explained by a decline in general cardiovascular risk factors, such as glycaemic status, hypertension or atherosclerosis.

We recently reported direct cardiac effects of EMPA [4]. EMPA lowers cytosolic Na^+^ ([Na^+^]_c_) and cytosolic Ca^2+^ ([Ca^2+^]_c_) concentrations, while increasing mitochondrial Ca^2+^ concentrations, through inhibition of the myocardial Na^+^/H^+^ exchanger (NHE) in isolated rabbit and rat ventricular cardiomyocytes. Both increased [Na^+^]_c_ and upregulated NHE activity have been shown to contribute to heart failure and diabetes [5–9]. We therefore hypothesised that these cardiac effects of NHE inhibition constituted a causal mechanism for the beneficial clinical effects of EMPA. It is not known whether NHE inhibition is a drug-specific effect of EMPA or a class effect of SGLT2i. Furthermore, molecular binding between SGLT2i and NHE has to our knowledge not been studied. Additionally, no data are available on the direct cardiac effects of SGLT2i on the healthy heart. We therefore studied cardiac NHE activity and [Na^+^]_c_ in mouse cardiomyocytes for the SGLT2i EMPA, dapagliflozin (DAPA) and CANA and performed docking studies to identify possible binding sites of SGLT2i on NHE. In addition, we studied whether there were direct cardiac haemodynamic and metabolic effects of these SGLT2i on the healthy intact heart.

## Methods

A detailed description of the methodology is available in the electronic supplementary material (ESM) Methods.

Animal handling of C57Bl/6NCrl male mice was in accordance with the Institutional Animal Care and Use Committee of the Academic Medical Center, University of Amsterdam, Amsterdam, the Netherlands, and was conducted following the Guide for the Use and Care of Laboratory Animals. Freshly isolated ventricular cardiomyocytes were subjected to 1 μmol/l EMPA, 1 μmol/l DAPA, 3 μmol/l CANA or vehicle (0.02% DMSO [vol./vol.]). These concentrations were based on the maximum plasma concentration of each drug found in vivo at clinically relevant doses [10]. NHE activity was quantified from recovery of pH following an acidifying NH_4_^+^ pulse with the use of seminaphtharhodafluor (SNARF) fluorescence. [Na^+^]_c_ was detected using the sodium-binding benzofuran isophthalate 1 (SBFI-1) fluorescent probe.

A homology model of the protein structure of human NHE-1 was prepared using a bacterial NHE protein structure as a template. Molecular docking studies were performed using the AutoDock Vina software package [11] to explore possible interactions between NHE and SGLT2i.

Langendorff constant-flow perfused mouse hearts were subjected to SGLT2i (at the same concentrations as mentioned above) for 30 min, during which cardiac haemodynamics were monitored. O_2_ consumption was determined (at *t* = 25 min following the start of drug infusion) from the difference between coronary influent and effluent O_2_ levels, and was normalised for heart dry weight and coronary flow. ATP and phosphocreatine (PCr) levels were spectrophotometrically determined in snap-frozen and freeze-dried hearts.

All data were tested for normality using the Kolmogorov–Smirnov test. Normally distributed data were statistically tested by one-way ANOVA with Dunnett’s post hoc tests and were presented as mean ± SD. Not normally distributed data (median, interquartile range), including data for cardiac O_2_ consumption and energetics of healthy hearts, were tested with the Kruskal–Wallis test; post hoc analysis was conducted with Mann–Whitney *U* tests and Bonferroni correction. All tests were carried out using the SPSS statistics 24 package (IBM SPSS, Armonk, NY, USA). A *p* value below 0.05 was considered statistically significant.

## Results

### SGLT2i can bind to NHE, block NHE activity and reduce [Na^+^]_c_ in cardiomyocytes

**Fig. 1:**
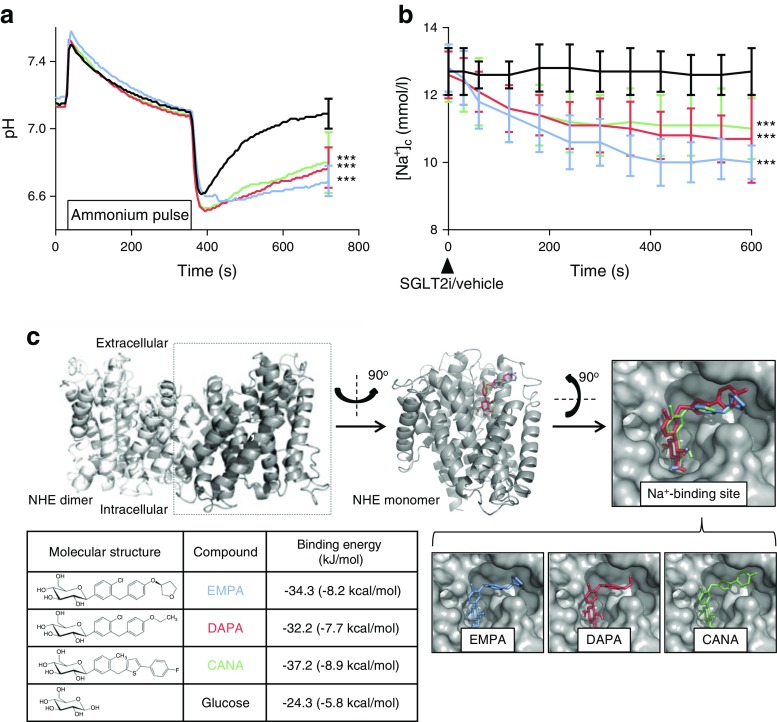
EMPA, CANA and DAPA inhibit NHE activity, reduce [Na^+^]_c_ and bind to the Na^+^-binding site of NHE-1. (**a**) SGLT2i inhibit NHE activity in cardiomyocytes. Recovery of the pH is a measure of NHE activity and was only seen in vehicle (black line). All three drugs blocked pH recovery, and thus NHE activation, significantly, compared with vehicle (***p < 0.001). (**b**) [Na^+^]_c_ was reduced in all three SGLT2i (***p < 0.001). Data are presented as mean ± SD and are derived from eight cells from four mouse hearts for each condition. (**c**) In silico analysis of SGLT2i binding to a homology model of NHE. All three SGLT2i efficiently bind to the Na^+^-binding pocket of NHE. Calculated binding affinities of SGLT2i are much higher than the negative control, glucose. EMPA is shown in blue, DAPA is shown in red and CANA is shown in green

We investigated whether SGLT2i exhibited similar effects on NHE by studying intracellular Na^+^ and pH. Figure 1a shows reduced recovery of intracellular pH after NH_4_^+^ pulse with all three SGLT2i (EMPA 6.69 ± 0.09, DAPA 6.77 ± 0.12 and CANA 6.80 ± 0.18 vs vehicle 7.09 ± 0.09; *p* < 0.001 for all three comparisons). Furthermore, [Na^+^]_c_ was reduced with all three SGLT2i (Fig. 1b) after 10 min (in mmol/l: EMPA 10.0 ± 0.5, DAPA 10.7 ± 0.7 and CANA 11.0 ± 0.9 vs vehicle 12.7 ± 0.7; *p* < 0.001 for all three comparisons).

Interactions between SGLT2i and NHE were explored using a homology model of NHE. The extracellular part of the protein model was probed to gain insight into the possible binding mode of SGLT2i to NHE. All three SGLT2i displayed high binding affinity to the extracellular Na^+^-binding site of NHE (in kJ/mol: EMPA −34.3, DAPA −32.2, CANA −37.2) and adopted similar binding conformation with the glucoside moiety oriented towards the Na^+^-binding site and the aglycone part of the inhibitors lining the side of the extracellular aperture (Fig. 1c). Binding of a glucose molecule to the NHE homology model showed that glucose bound in an identical orientation to the glucoside part of the SGLT2i, but with a reduced affinity of −24.3 kJ/mol, illustrating the importance of the hydrophobic part of the SGLT2i to ensure efficient binding.

Combined, these data show that EMPA, DAPA and CANA inhibit NHE activity through binding to the Na^+^-binding site of NHE-1. Secondary to this, they reduce [Na^+^]_c_ in isolated cardiomyocytes.

### EMPA and CANA induce vasodilation in intact hearts

**Fig. 2:**
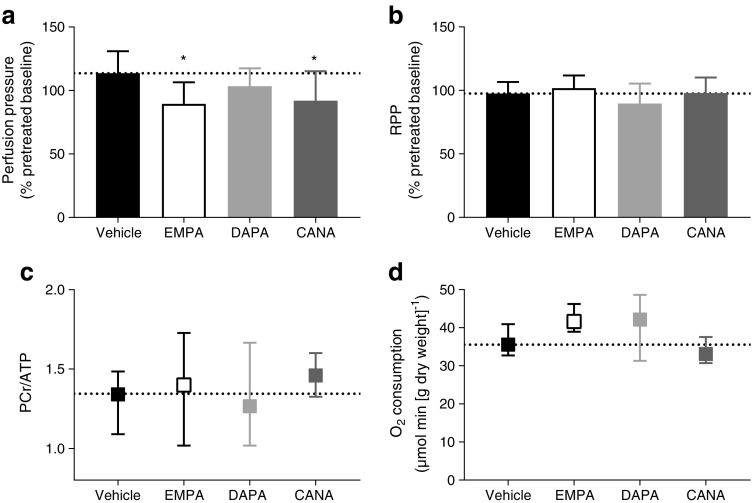
EMPA and CANA induced coronary vasodilation in the healthy intact heart. Hearts were perfused for 30 min with vehicle (0.02% DMSO; *n* = 15, black bars), 1 μmol/l EMPA (*n* = 9, white bars), 1 μmol/l DAPA (*n* = 10, light grey bars) or 3 μmol/l CANA (*n* = 10, dark grey bars). (**a**) EMPA and CANA significantly reduced perfusion pressure after 30 min (*p < 0.05). (**b**) SGLT2i administration did not change rate pressure product (RPP) and (**c**) PCr/ATP (vehicle *n* = 13, EMPA *n* = 8, DAPA *n* = 10, CANA *n* = 10). Values at *t* = 30 min are normalised to values at *t* = 0 min from the same condition. (**d**) No changes were observed for O_2_ consumption for all three SGLT2i (vehicle *n* = 12, EMPA *n* = 8, DAPA *n* = 8, CANA *n* = 10). The dashed line in each graph represents the mean of vehicle condition. Data for perfusion pressure (**a**) and RPP (**b**) are presented as mean ± SD; data of PCr/ATP (**c**) and O_2_ consumption (**d**) are presented as median, interquartile range

Perfusion pressure at *t* = 0 min was not significantly different between groups (in mmHg: vehicle 79.4 ± 2.7, EMPA 79.9 ± 2.4, DAPA 79.3 ± 4.1, CANA 79.3 ± 2.5). After 30 min following drug administration, EMPA and CANA resulted in vasodilation, compared with vehicle, in isolated constant-flow perfused mouse hearts (vehicle vs EMPA *p* < 0.05, CANA *p* < 0.05, DAPA *p* = 0.410) (Fig. 2a). Furthermore, cardiac functional performance (rate pressure product), cardiac energetic status (PCr/ATP) and O_2_ consumption were unchanged after administration of the SGLT2i (Fig. 2b–d, respectively).

## Discussion

This study demonstrates for the first time that (1) EMPA, DAPA and CANA inhibit cardiac NHE and reduce [Na^+^]_c_ in cardiomyocytes; (2) EMPA, DAPA and CANA display high binding affinity to the extracellular Na^+^-binding site of NHE; (3) EMPA and CANA cause vasodilation in the isolated healthy heart. The effects of EMPA, DAPA and CANA on cardiac [Na^+^]_c_ through NHE inhibition can therefore be considered a common class effect of SGLT2i. Knowing that elevated [Na^+^]_c_ is a common denominator and driver of diabetes and heart failure [9, 12], we propose that the potential of SGLT2i to lower cardiac [Na^+^]_c_ contributes to reduced heart failure-related hospitalisation, as observed in the BI 10773 (Empagliflozin) Cardiovascular Outcome Event Trial in Type 2 Diabetes Mellitus Patients (EMPA-REG OUTCOME) trial and the Canagliflozin Cardiovascular Assessment Study (CANVAS) [2, 3]. Further studies are necessary to examine this hypothesis.

### EMPA, CANA and DAPA all inhibit NHE activity, reduce [Na^+^]_c,_ and show high binding energy with NHE

Our experiments examining NHE activity and [Na^+^]_c_ in cardiomyocytes showed that the inhibitory effect on cardiac NHE is not specific to EMPA but extends to other SGLT2i. In our previous work [4], we showed that EMPA treatment not only lowers [Na^+^]_c_ but reduces [Ca^2+^]_c_ and increases mitochondrial Ca^2+^. Since DAPA and CANA also inhibit NHE activity and reduce [Na^+^]_c_, we postulate that these SGLT2i reduce [Ca^2+^]_c_ and increase mitochondrial Ca^2+^ similar to EMPA, and ultimately optimise cardiac mitochondrial function and energetics. Because both [Na^+^]_c_ and NHE activity are increased in diabetic and failing hearts, SGLT2i should have an even stronger effect on these in diseased cardiomyocytes. Furthermore, preclinical studies with known NHE inhibitors have clearly shown reductions in the development of hypertrophy and heart failure [13, 14], which support NHE inhibition and its consequential [Na^+^]_c_-lowering as a potential class effect of SGLT2i to combat heart failure.

SGLT2i are targeted to SGLT2 by the glucosyl part of the molecule, while binding affinity is determined by the attached hydrophobic moiety. Our docking studies indicate that the hydrophilic glucosyl part of the SGLT2i orients towards the hydrophilic Na^+^-binding site. The composition of the hydrophobic aglycone part of the SGLT2i appears to be an important determinant in their binding to NHE. The NHE molecule exists in a low- and high-affinity form for intracellular protons, regulated by pH and mitogens [15]. This conformational heterogeneity may be essential in SGLT2i binding, especially in disease states where NHE activity threshold is shifted towards its high-affinity form.

### Vasodilation by SGLT2i in the healthy heart

Our data on isolated mice hearts revealed a direct vasodilation effect of EMPA and CANA, but not DAPA, at constant glucose concentration. Oelze et al [16] have previously shown that EMPA normalised endothelial function in aortic rings from streptozotocin-induced rat models of diabetes, an effect that was also detected with ipragliflozin in a similar mouse model [17]. However, because EMPA treatment in these studies also caused a large reduction in plasma glucose levels, it is impossible to interpret these data towards EMPA exerting direct vascular effects. Wang et al [18] reported that NHE activation in hyperglycaemic endothelial cells led to increased intracellular Ca^2+^ and reduced endothelial nitric oxide synthase levels and impaired relaxation of aortic rings from streptozotocin-induced rat models of diabetes, while NHE inhibition abolished these effects. Assuming that NHE inhibition by SGLT2i also occurred in other cells than cardiomyocytes in our intact heart experiments, vasodilation by SGLT2i may therefore be related to lowering of [Ca^2+^]_c_ in endothelial cells or vascular smooth muscle cells after NHE inhibition. Finally, no changes were observed for cardiac workload, energetic status and metabolic function in healthy hearts. The functional and energetic status of healthy hearts was already optimal and could not be improved by treatment with SGLT2i. Interestingly, a preliminary study in *db*/*db* mice found that EMPA administration acutely improved PCr/ATP [19]. In our experiments, we did notice a non-significant trend of increased O_2_ consumption in EMPA-treated hearts (*p* = 0.054), which may possibly indicate increased activation of mitochondrial energy metabolism.

We cannot explain why DAPA did not significantly induce vasodilation in healthy hearts. The non-significant results for DAPA in relation to vasodilation could in part be explained by the relatively low sample size. Here, we only studied the direct effects of SGLT2i for 30 min in isolated hearts. Chronic cardiac effects of SGLT2i may be studied in the future in in vivo models to translate and understand drug effects in individuals who use SGLT2i daily. Another limitation of this study is the lack of a diabetic model to investigate direct cardiac effects of SGLT2i. Nonetheless, the results in healthy cells and hearts suggest that these direct effects of SGLT2i may happen regardless of diabetes, opening the possibility to explore SGLT2i in other cardiac diseases where increased NHE activity is a driver of the disease, such as heart failure and hypertrophy. Thus, future research should also examine the effects of SGLT2i on cardiac physiology and metabolism in diabetic and failing hearts.

In conclusion, EMPA, DAPA and CANA all exhibit direct cardiac effects through NHE inhibition and [Na^+^]_c_ reduction. EMPA and CANA, but not DAPA, induce coronary dilation of the intact heart.

## Electronic supplementary material

ESM (PDF 104 kb)

## Abbreviations

[Ca^2+^]_c_: Cytosolic Ca^2+^ concentration
CANA: Canagliflozin
DAPA: Dapagliflozin
EMPA: Empagliflozin
[Na^+^]_c_: Cytosolic Na^+^ concentration
NHE: Na^+^/H^+^ exchanger
PCr: Phosphocreatine
SGLT2: Sodium–glucose cotransporter 2
SGLT2i: Sodium–glucose cotransporter 2 inhibitor(s)
