# Supplementary material for: Class effects of SGLT2 inhibitors in mouse cardiomyocytes and hearts: inhibition of Na+/H+ exchanger, lowering of cytosolic Na+ and vasodilation
Source: Diabetologia. 2017 Dec 2;61(3):722–6. doi: 10.1007/s00125-017-4509-7 (PMC6448958; doi:10.1007/s00125-017-4509-7)
Supplement: Supplementary file 1 — (PDF 104 kb) [file 125_2017_4509_MOESM1_ESM.pdf]

Electronic supplementary material (ESM) Methods

Animals

C57Bl6/NCrl male mice (8-12 weeks old, Charles River, Lyon, France) were housed with a maximum of 7 mice per cage for at least one week, with a 12 hours day/night cycle, and food (Teklad global 16% protein rodent diet (Envigo, Indianapolis, IN, US)) and drinking water ad libitum. Mice were injected intraperitoneally with heparin (15 mU) and anesthetized with 138±2 mg/kg body weight S(+)-ketamine and 0.220±0.003 mg/kg body weight dexmedetomidine. Anaesthetic depth was tested with multiple toe pinches. Mice were intratracheally ventilated with 50% O_2 and 50% N_2, prior to in-chest aortic cannulation for immediate perfusion of hearts.

NHE activity and $[Na^+]_c$ measurements in isolated cardiomyocytes

Cardiomyocytes were isolated from healthy mouse hearts and $[Na^+]_c$ and NHE were measured as described previously [1, 2]. $[Na^+]_c$ was determined fluorometrically with the sodium-binding benzofuran isophthalate 1 (SBFI-1) fluorescence probe (Abcam, Cambridge, United Kingdom; 340 nm excitation, 410/590 nm emission). Myocytes were incubated with 10 µmol/l SBF1-AM for 30 min at 37 °C, washed twice with fresh HEPES solution and incubated for another 15 min for complete de-esterification. Subsequently, myocytes were seeded on 0.1g/L poly-S-lysine treated cover slips and placed in a temperature controlled (37 °C) perfusion chamber (height 0.4mm, diameter 10mm, volume 30µl), which was staged on an inverted fluorescence microscope (Nikon Diaphot, Tokyo, Japan). Quiescent single myocytes were field stimulated at 2 Hz frequency and dual wave length emission wave length was measured during 100 ms light flashes at 1 kHz, and corrected for the fluorescence of unloaded myocytes. NHE activity was measured with seminaphtharhodafluor (SNARF) fluorescence (Molecular probes, Eugene, USA; 580/640 nm emission; 515 nm excitation) after NH_4^+ pulse. Myocytes were treated with 10 µmol/l SNARF-AM for 30 min at 37 °C, after which they were first superfused with HEPES buffer for 1 min and next with HEPES containing 20 mmol/l NH_4Cl for 10 min, together with the treatment agent (EMPA, DAPA, CANA or vehicle). Recovery after NH_4^+-pulse is a measure of NHE activity. 5 mmol/l EMPA, 5 mmol/l DAPA or 15 mmol/l CANA (all from MedChem Express, New Jersey, USA) in 100% DMSO was added to HEPES buffer to a final concentration of 1 µmol/l EMPA, 1 µmol/l DAPA or 3 µmol/l CANA in 0.02% DMSO [vol./vol.]. $[Na^+]_c$ and NHE activity are derived from n=8 cells per assay from 4 mouse hearts for each condition. Experimenters were not blinded to group assignment and outcome assessment and randomisation was not applied in the cardiomyocyte and heart experiments. However, the sequence of each drug tested was

randomised with each biological replicate to correct for variation occurring throughout the day.

In silico NHE-1 docking studies

A homology model of the human NHE-1 (uniprot P19634, gene SLC9A1) was generated using the Phyre2 homology modelling portal [3]. In the top prediction, a large part of the NHE-1 transporter (50% sequence coverage, amino acid residues 100-513) was modelled with 100% confidence using the bacterial sodium-proton antiporter from Methanocaldococcus Jannaschii (PDB 4CZB) as a template. Even though inter-species sequence identity is relatively low (usually only 15-20%), structural helical elements are strongly conserved in this protein family, allowing for the generation of a high-confidence homology model. The modelled region (roughly 100-550) is the trans-membrane domain that contains 12 transmembrane helices.

Next, ligand docking studies were performed on the homology model using AutoDock Vina [4]. This software suite allows for flexible docking of drug-like compounds; different docking regions of the target molecule can be selected. A .pdbqt file of the predicted protein model was prepared in AutoDockTools [5]. Initial studies in which compounds were docked onto the complete model of the NHE protein showed a clear binding preference for the extracellular Na^+-binding pocket. To compare relative binding affinities, a more targeted approach was chosen and specific binding to the Na^+-binding pocket was evaluated. Settings for these runs were as follows: Grid box: x=y=z=30; center values: x=76.8, y=47.5, z=120.8; exhaustiveness 12; num modes 10. Multiple runs were performed (usually > 20) to allow for identification of the correct binding modes.

Isolated heart experiments

Hearts were perfused with Krebs Henseleit buffer (KHB, composition (in mmol/l): 118 NaCl, 4.7 KCl, 1.2 $MgSO_4$, 1.2 KH_2PO_4, 25 $NaHCO_3$, 0.5 EDTA, 2.25 $CaCl_2$, 7 D-glucose, 0.5 L-glutamine, 1 lactate and 0.1 pyruvate) containing 0.1% albumin [w./vol.] and 50 mU/L insulin [6]. Hearts were stabilized for 20 min in a 37 ^oC KHB-filled bath until perfusion pressure reached 75-85 mmHg, left ventricular (LV) diastolic pressure was 3-5 mmHg and LV developed pressure was at least 80 mmHg. Experiments were performed with 15 hearts for DMSO, 10 for EMPA, 10 for DAPA and 10 for CANA. Exclusion of samples occurred due to unsuccessful aortic cannulation (n=1 of EMPA group), insufficient sample for energetic measurements (n=2 of DMSO group, n=1 of EMPA group) or failed sampling of pulmonary effluent (n=3 of DMSO group, n=1 of EMPA group, n=2 of DAPA group). CANA (15 mmol/l), EMPA and DAPA (both 5 mmol/l) stocks in 100% DMSO were diluted with distilled water to

300 µmol/l and 100 µmol/l respectively, which were subsequently added to KHB via controlled pump flow (Harvard pump apparatus; Holliston, MA, USA). The pump was set 100 times slower than coronary flow, thereby getting a concentration of 1 µmol/l EMPA, 1 µmol/l DAPA or 3 µmol/l CANA in the hearts. Administration occurred for 30 min during which physiological parameters were monitored. Myocardial O_2 consumption was determined by blood gas analyses (RapidPoint Siemens blood gas apparatus from Siemens Healthcare Nederland B.V., Den Haag, The Netherlands) of coronary effluent samples taken at t=25 min subtracted from influent samples taken after heart excision. At t=30 min, hearts were snap frozen into liquid nitrogen and stored in -80 ^{o}C until further processing. Hearts were then freeze-dried overnight, and subjected to acidic treatment with 4% perchloric acid [w./vol.] to block enzymatic activity and subsequently neutralized to pH 7.0.

Spectrophotometric analysis of PCr and ATP

ATP and PCr were measured by inducing a set of reactions (illustrated in ESM Fig. 1) where all compounds were present in excess, except for ATP and PCr [7]. Conversion of $NADP^+$ to NADPH, shown in reaction 3, will thus measure [ATP] and [PCr] in tissue. For this, 120 mmol/l glucose and 105 U/mL creatine kinase (only for PCr determination; Roche, Basel, Switserland) were dissolved in CK kit solution 2 (Roche/Hitachi). Samples were added to reagent solution with a 1:11 dilution factor and incubated for 10 min at 30 ^{o}C in dark. Absorbance was measured photospectrometrically (Specord 50 from Analytik Jena AG, Jena, Germany) at 340 nm and converted to concentration using the extinction coefficient of NAPDH (6.22 mmol l^{-1} cm^{-1}).Finally, sample absorbance was corrected for background absorbance of reagent solution and samples, weight of samples and glucose-6-phosphate levels in samples.

Reaction 1: $PCr + ADP \xrightarrow{CK} Creatine + ATP$

Reaction 2: $ATP + Glucose \xrightarrow{HK} G6P + ADP$

Reaction 3: $G6P + NADP^{+} \xrightarrow{G6PDH} 6\text{-}PGN + NADPH + H^{+}$

ESM Fig 1 Principle of spectrophotometric reactions to quantify tissue PCr and ATP levels. ATP levels were determined by stimulation of reaction 2 and 3, PCr levels were determined through reaction 1 to 3. CK creatine kinase, HK hexokinase, G6P glucose-6-phosphate, G6PDH glucose-6-phosphate dehydrogenase, 6-PGN 6-Phosphogluconic acid.

References

1. ter Welle HF, Baartscheer A, Fiolet JW, Schumacher CA (1988) The cytoplasmic free energy of ATP hydrolysis in isolated rod-shaped rat ventricular myocytes. J Mol Cell Cardiol, 20, 435–41.

2. Baartscheer A, Schumacher CA, Wüst RCI et al (2017) Empagliflozin decreases myocardial cytoplasmic Na^+ through inhibition of the cardiac Na^+/H^+ exchanger in rats and rabbits. Diabetologia, 60, 568–573

3. Kelley LA, Mezulis S, Yates CM, Wass MN, Sternberg MJE (2015) The Phyre2 web portal for protein modeling, prediction and analysis. Nat Protoc, 10, 845–858

4. Trott O, Olson A (2010) AutoDock Vina: improving the speed and accuracy of docking with a new scoring function, efficient optimization and multithreading. J Comput Chem, 31, 455–461

5. Sanner MF (1999) Python: A Programming Language for Software Integration and Development. J Mol Graph Model, 17, 57–61

6. Smeele KMA, Southworth R, Wu R et al (2011) Disruption of hexokinase II-mitochondrial binding blocks ischemic preconditioning and causes rapid cardiac necrosis. Circ Res, 108, 1165–1169

7. Fiolet JWT, Baartscheer A, Schumacher CA, Coronel R, ter Welle HF (1984) The change of the free energy of ATP hydrolysis during global ischemia and anoxia in the rat heart. Its possible role in the regulation of transsarcolemmal sodium and potassium gradients.J Mol Cell Cardiol, 16, 1023–1036
